# Glycaemic variability is associated with all-cause mortality in COVID-19 patients with ARDS, a retrospective subcohort study

**DOI:** 10.1038/s41598-022-13816-8

**Published:** 2022-06-14

**Authors:** Bojan Hartmann, Marlo Verket, Paul Balfanz, Niels-Ulrik Hartmann, Malte Jacobsen, Julia Brandts, Michael Dreher, Nils Kossack, Dennis Häckl, Nikolaus Marx, Dirk Müller-Wieland

**Affiliations:** 1 Department of Cardiology, Angiology and Intensive Care Medicine, University Hospital Aachen, Aachen, Germany; 2 Department of Pneumology and Intensive Care Medicine, University Hospital Aachen, Aachen, Germany; 3 WIG2 – Scientific Institute for Health Economics and Health System Research, Leipzig, Germany; 4 Faculty of Economics and Management Science, University Leipzig, Leipzig, Germany; 5 Department of Cardiology, Angiology and Intensive Care Medicine, Medical Clinic I, University Hospital Aachen, Pauwelsstraße 30, 52074 Aachen, Germany

**Keywords:** Biomarkers, Risk factors, Diabetes complications, Type 2 diabetes, Respiratory distress syndrome, Prognostic markers

## Abstract

There is high mortality among intensive care unit (ICU) patients with acute respiratory distress syndrome (ARDS) caused by coronavirus disease (COVID-19). Important factors for COVID-19 mortality are diabetes status and elevated fasting plasma glucose (FPG). However, the effect of glycaemic variability on survival has not been explored in patients with COVID-19 and ARDS. This single-centre cohort study compared several metrics of glycaemic variability for goodness-of-fit in patients requiring mechanical ventilation due to COVID-19 ARDS in the ICU at University Hospital Aachen, Germany. 106 patients had moderate to severe ARDS (P/F ratio median [IQR]: 112 [87–148] mmHg). Continuous HRs showed a proportional increase in mortality risk with daily glycaemic variability (DGV). Multivariable unadjusted and adjusted Cox-models showed a statistically significant difference in mortality for DGV (HR: 1.02, (P) < 0.001, LR(P) < 0.001; HR: 1.016, (P) = 0.001, LR(P) < 0.001, respectively). Kaplan–Meier estimators yielded a shorter median survival (25 vs. 87 days) and a higher likelihood of death (75% vs. 31%) in patients with DGV ≥ 25.5 mg/dl (*P* < 0.0001). High glycaemic variability during ICU admission is associated with significant increase in all-cause mortality for patients admitted with COVID-19 ARDS to the ICU. This effect persisted even after adjustment for clinically predetermined confounders, including diabetes, median procalcitonin and FPG.

## Introduction

Acute respiratory distress syndrome (ARDS) caused by coronavirus disease (COVID-19) has been associated with a high mortality rate for patients in the intensive care unit (ICU). Recently, a pooled global mortality rate of patients who have been admitted with COVID-19 associated ARDS to ICU is estimated to be 39% (95% CI 23–56%)^1^. This high mortality in COVID-19 with ARDS highlights the importance of identifying clinical features and various biomarkers as predictors of poor disease outcomes over time. Furthermore, type 2 diabetes (T2D), obesity, hypertension, and cardiovascular diseases increase the severity and fatal outcome in patients with COVID-19 associated ARDS^2,3^.

Fasting plasma glucose (FPG) level and HbA1c at admission has been discussed as a predictor for mortality outcome in patients with COVID-19 and ARDS. Previous studies suggest that elevated glucose levels on admission^4,5^ and plasma glucose fluctuations in the early phase^6^ of hospitalisation in COVID-19 patients predict adverse outcomes regardless of diabetes status. Recently, a multi-centre study^7^ in Italy demonstrated that glucose levels of patients who were admitted with COVID-19 to the ICU were significantly higher in non-survivors than in survivors. However, when considering the effect of FPG, these studies have concentrated on the absolute values of FPG and not the variability of glucose over time.

To date, studies mainly focused on hyperglycaemia on admission, while glycaemic variability throughout ICU admission has not been sufficiently studied. In this report, we evaluate the effect of glucose level parameters and glycaemic variability on patient survival, specifically in patients admitted to the ICU with ARDS due to COVID-19.

## Materials and methods

### Study population

**Figure 1:**
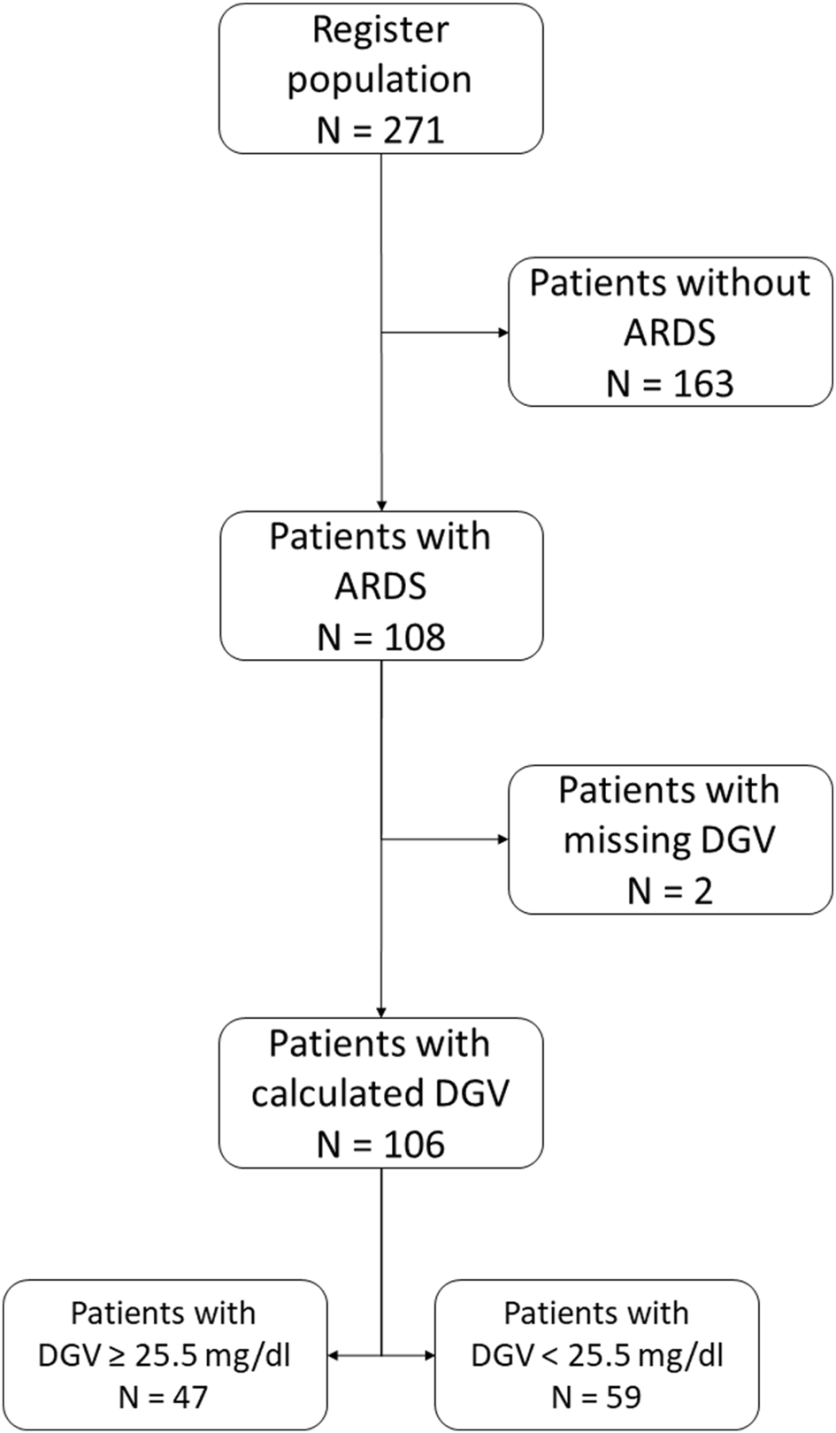
Flowchart of patient enrolment. The register population represents all patients included in the COVAS cohort. A total of 106 out of 271 patients were enrolled in this subgroup analysis. *ARDS* acute respiratory distress syndrome; *DGV* daily glycaemic variability.

106 patients with laboratory-confirmed COVID-19 who were admitted to the ICU for treatment of ARDS at University Hospital Aachen, Germany, were recruited to this retrospective subcohort study. 59 of these patients were published^8,9^ previously in respect to a single-centre cohort study, COVAS. Patients in the present study were admitted between February 24, 2020, until May 15, 2021 and fulfilled the following criteria (Fig. 1). Inclusion criteria for the subcohort were a positive respiratory SARS-CoV-2 PCR result and admission to the ICU requiring mechanical ventilation due to COVID-19 and ARDS. Exclusion criteria of this study were lack of consent, positive PCR result or age of majority, as well as pregnancy or inability to legally give consent. In order to calculate the variability of FPG levels, we excluded patients who did not have at least 3 days of consecutive glucose measurements during their admission to the ICU.

Since University Hospital Aachen is designated a tertiary care facility, patients with minor or mild severity were triaged by the emergency services towards other regional hospitals. Thus, the present study includes a significant number of patients with severe clinical course from other regional hospitals, who were either previously screened for ECMO or other high-end treatment methods.

All patients gave their written informed consent before participating in the COVAS study, which complies with the Declaration of Helsinki. Study approval was acquired by the ethics committee at the Faculty of Medicine of RWTH Aachen University (EK080/20). This trial has been retrospectively registered in the German Clinical Trials Register (DRKS00027106).

Based on national guidelines^10^ and internal standards of operation, all patients with an FPG above 180 mg/dl were titrated to a FPG target of 150 mg/dl using continuous insulin infusion during ICU admission.

### Definitions of study parameters, exposures and outcomes

ARDS was defined according to the Berlin definition^11^. Comorbidities were defined as conditions that were known before hospital admission. Likewise, previous medication included any medication prescribed before admission to our hospital.

Baseline vital parameters are characterized as the first available measurements after ICU admission. Respiratory disease was defined as a composite of bronchial asthma, chronic obstructive pulmonary disease, obstructive sleep apnoea syndrome and pulmonary malignancy. Moreover, composite heart disease is a composite of arterial hypertension, atrial fibrillation, coronary artery disease, heart failure and previous myocardial infarction. History of T2D was specified either by a previously known T2D diagnosis, diabetes medication at time of hospital admission or HbA1c at admission of ≥ 6.5% (48 mmol/mol).

For outcome measures, the primary endpoint was defined as all-cause mortality during ICU admission. As exposures, high and low glycaemic variabilities during ICU admission were defined as daily glycaemic variability (DGV) ≥ 25.5 mg/dl and DGV < 25.5 mg/dl. To determine this cut-off for DGV, we fitted a regression tree model (25.5 mg/dl) and compared it to a cut-off based on a hazard ratio of 1 derived from a Cox-PH model, which was adjusted for age, sex and history of T2D (31 mg/dl). The regression tree-based cut-off demonstrated a higher AUC (0.729 vs. 0.689) in 30-day survivalROC curves, therefore we used the cut-off DGV value of 25.5 mg/dl in further models and testing rather than the Cox-PH based cut-off of DGV 31 mg/dl.

### Data acquisition

We collected symptoms on admission, co-morbidities and previous medication either per interview/questionnaire in alert patients or per admission/discharge documents from our emergency department and previous hospitals. Vital parameters were acquired immediately on the first day of ICU admission. On subsequent days, we recorded the worst daily value, in the context of shock and/or respiratory failure. All data was manually retrieved from our EHR software, which automatically transfers ventilation parameters at set intervals from the ventilator to the patient’s electronic health record. In order to reduce confounders in ventilation parameters due to this automated process and initially extreme ventilation parameters, we intentionally omitted the first four hours of ventilation parameters after admission and intubation to allow the staff to properly configure the ventilator according to the patient’s requirements at the time.

PCR results were acquired by quantitative real-time polymerase-chain-reaction (PCR). Diagnosis of COVID-19 was established by positive respiratory PCR from either a throat swab or tracheal fluid in awake patients and bronchoalveolar lavage (BAL) in intubated patients. Respiratory PCR was repeated on days 7 and 14 of admission. Additionally, BAL, serum, stool and urine samples were tested for bacterial, fungal and viral pathogens, including Legionella pneumophila and Streptococcus pneumoniae antigens as well as SARS-CoV-2. All patients received daily routine laboratory tests including glucose levels between 03:00 – 05:00 AM.

### Statistical analysis

All statistical analysis was performed in R version 4.1.2^12^ using packages ggplot2 (version 3.3.5)^13^ for scatter plots, tangram (0.7.1)^14^ for tables and Rmarkdown for text. The characteristics were described as median (IQR) for continuous and percentages for categorical variables. Categorical parameters were compared by Fisher’s Exact Test and continuous parameters by Kruskal–Wallis test. Statistical significance was determined as a *p*-value below 0.05. We opted not to do any parameter imputation for missing values.

In order to select a suitable metric to evaluate fasting plasma glucose variability, we first compared established parameters: standard deviation (SD), Neuman’s (root) mean square of successive differences (MSSD and rMSSD), bias corrected coefficient of variation (CoefVar) and median of the absolute difference between successive values (DGV, daily glycaemic variability). In order to compute DGV, we first calculated the absolute differences of FPG (ΔFPG) for consecutive days, where FPG_day_ represents the fasting plasma glucose of the current day and FPG_day+1_ the fasting plasma glucose of the following day (Eq. 1):

1 $$\Delta FP{G}_{day}=\left|FP{G}_{day}-FP{G}_{day+1}\right|$$

Then, we calculated DGV as the median of all (ΔFPG) values for each patient.

We calculated MSSD and rMSSD using the psych package (version 2.1.9)^15^ and CoefVar using the implementation provided by the DescTools package (version 0.99.44)^16^.

The cut-off DGV was estimated by regression tree analysis using rpart (version 4.1.16)^17^. Through rms (version 6.2.0)^18^, smooth hazard ratios and survival analysis were examined in Cox-proportional-hazard (Cox-PH) regression models, which were compared by likelihood ratio (LR) test, Akaike information criterion (AIC) and Concordance Index (C-Index).

All Cox-PH models were tested for the proportional hazard assumption as well as for collinearity utilising the variance inflation factor (vif function rms^18^ package). While recommendations vary, in accordance with most studies, we defined a VIF < 5 as acceptable.

Before analysis and based on clinical judgment we selected the following confounders for adjustment of our final Cox-PH models: age, sex, BMI, history of type 2 diabetes (T2D), dialysis during admission, dexamethasone treatment, median procalcitonin (PCT) and FPG during ICU admission. To reduce overadjustment of the models, we removed BMI and dialysis during admission from the final model. For this model we additionally used Firth’s penalized maximum likelihood bias reduction method, provided by the coxphf (version 1.13.1)^19^ package.

To evaluate the accuracy of the outcome-based cut-offs, we compared the AUC in 30-day survival models using the implementation of survivalROC (version 1.0.3)^20^.

Utilizing survminer’s (version 0.4.9)^21^ ggforest function, forest plots were created. Furthermore, Kaplan–Meier estimator was calculated with the survival (version 3.2.13)^22^ package and plotted with survminer’s (version 0.4.9)^21^ ggsurvplot function, which compared survival curves and computed *p*-values using the log-rank test.

### Ethics approval and consent to participate

Study approval was acquired by the ethics committee at the Faculty of Medicine of RWTH Aachen University (EK080/20). The present research complies with the Declaration of Helsinki.

## Results

A total of 106 patients with ARDS caused by COVID-19 were included in this retrospective analysis and 53 (50%) of patients were defined as survivors. Characteristics in this cohort are summarized in Table 1. The majority were diagnosed with moderate to severe ARDS on admission (P/F ratio median [IQR]: 112 [87–148] mmHg). In total 32 (30%) had a previous T2D diagnosis and 10 patients with HbA1c ≥ 6.5% (48 mmol/mol) had newly diagnosed T2D. We identified 58 (54%) patients with hyperglycaemia (FPG > 140 mg/dl) on admission to the ICU, of which 33 (56%) had no prior diagnosis of T2D.

**Table 1:** Characteristics of patients admitted with COVID-19 and ARDS to the Intensive Care Unit.

	N	Overall	DGV < 25.5 mg/dl	DGV ≥ 25.5 mg/dl	*P* value
		(N = 106)	(N = 59)	(N = 47)	
Age (years)	106	63.0 (57.0–71.0)	62.0 (55.3–69.0)	65.0 (58.2–72.8)	0.12
Female sex	106	34 (32·1%)	18 (30·5%)	16 (34·0%)	0.83
Systolic blood pressure (mmHg)	106	97.0 (84.0–104.0)	100.0 (87.2–105.8)	94.0 (83.0–101.8)	0.09
Diastolic blood pressure (mmHg)	106	51.5 (44.0–60.0)	54.0 (45.0–61.0)	49.0 (42.2–56.7)	0.05
Heart rate (1/min)	106	100.5 (86.0–116.0)	98.0 (84.3–112.8)	102.0 (90.3–118.0)	0.21
Temperature (°C)	106	37.8 (37.2–38.4)	38.1 (37.3–38.6)	37.6 (36.9–38.1)	** < 0.05**
Respiratory Rate (1/min)*	104	26.0 (22.0–29.6)	26.0 (22.0–30.0)	25.0 (21.0–27.3)	0.40
pH	95	7.4 (7.3–7.4)	7.4 (7.3–7.5)	7.3 (7.3–7.4)	0.16
Arterial oxygen saturation (%)	95	94.6 (90.9–96.9)	94.5 (91.3–96.7)	94.9 (90.2–97.5)	0.57
Arterial oxygen partial-pressure (mmHg)*	95	75.8 (60.9–92.9)	75.2 (60.9–90.5)	80.8 (61.3–101.8)	0.38
Arterial CO2 partial-pressure (mmHg)*	95	46.5 (35.5–60.4)	46.3 (36.7–59.1)	46.5 (34.0–63.2)	0.90
PEEP (cm H2O)*	98	13.0 (10.0–15.0)	13.0 (10.0–15.0)	13.0 (9.0–15.0)	0.69
Tidal volume per kg IBW (ml/kg)*	97	5.4 (4.2–6.5)	5.3 (4.0–6.1)	6.0 (4.6–6.8)	0.06
P/F ratio*	103	112.0 (86.2–148.0)	107.5 (85.9–145.1)	117.0 (87.0–163.7)	0.25
Ventilation Ratio*	87	1.8 (1.1–2.3)	1.6 (1.0–2.2)	1.8 (1.3–2.4)	0.33
BMI (kg/m^2^)	106	29.4 (26.3–33.8)	28.4 (26.3–31.1)	31.2 (27.5–38.1)	**0.01**
Composite respiratory disease	106	33 (31·1%)	17 (28·8%)	16 (34·0%)	0.67
Composite heart disease	106	34 (32·1%)	13 (22·0%)	21 (44·7%)	**0.02**
Hypertension	106	69 (65·1%)	34 (57·6%)	35 (74·5%)	0.10
Atrial fibrillation	106	13 (12·3%)	4 (6·8%)	9 (19·1%)	0.07
Coronary artery disease	106	25 (23·6%)	10 (16·9%)	15 (31·9%)	0.11
Myocardial infarction	106	15 (14·2%)	6 (10·2%)	9 (19·1%)	0.26
Fasting plasma glucose (mg/dl)	106	144.0 (116.0–189.1)	133.0 (106.2–161.7)	167.0 (131.3–224.3)	** < 0.01**
HbA1c (%)	87	6.1 (5.7–6.6)	6.0 (5.5–6.2)	6.4 (5.9–7.2)	** < 0.01**
Leukocytes (/µl)	106	10.0 (7.4–13.6)	10.0 (7.7–13.5)	9.7 (7.1–14.5)	0.96
Lymphocytes (/µl)	90	0.7 (0.3–1.1)	0.7 (0.3–1.1)	0.7 (0.5–1.0)	0.92
D-dimer (µg/l)	79	2252.0 (1237.8–7168.5)	3525.0 (1389.5–7244.5)	1614.5 (755.6–5688.1)	0.09
Ferritin (ng/ml)	56	1389.5 (823.9–2641.0)	1690.0 (1054.0–2792.7)	1020.5 (662.2–2057.8)	** < 0.05**
CRP (high-sense) (mg/l)	97	169.0 (113.6–287.3)	172.7 (131.4–275.4)	160.0 (86.8–300.7)	0.50
Procalcitonin (ng/ml)	106	0.5 (0.2–1.7)	0.4 (0.2–1.2)	0.6 (0.2–1.9)	0.31
IL-6 (pg/ml)	84	142.9 (77.9–325.5)	133.1 (67.0–338.0)	160.9 (95.1–294.3)	0.54

Numerical parameters are expressed as median (IQR) and categorical as N (%). The N column represents the number of non-missing values in each row to the right. *P*-values for differences between the two groups were tested using Fisher’s exact test for categorical variables and Kruskal–Wallis test for continuous variables. Bold indicates significant differences (*P* < 0.05). Parameters denoted with * represent the worst value on the day of admission. Composite heart disease is defined as atrial fibrillation, coronary artery disease, hypertension, heart failure or history of myocardial infarction. Composite respiratory disease was defined as a composite of bronchial asthma, chronic obstructive pulmonary disease, obstructive sleep apnoea syndrome and pulmonary malignancy.

We included 3147 glucose measurements in total in our analysis, each patient had a median [IQR] of 22 [12–41] measurements.

Utilizing a Cox-PH model (adjusted for age, sex and history of T2D) for FPG on admission as a continuous covariate, we found that high FPG on admission is a predictor of mortality. This model showed statistical significance for FPG and the model (HR: 1.00 [95% CI 1.00–1.01], Fasting plasma glucose (mg/dl) (P) < 0.001, LR(P) = 0.002) and demonstrated a linear increase in HR with admission FPG. In contrast HbA1c failed to show statistical significance in an equally adjusted model and additionally failed the proportional hazards assumption test.

**Figure 2:**
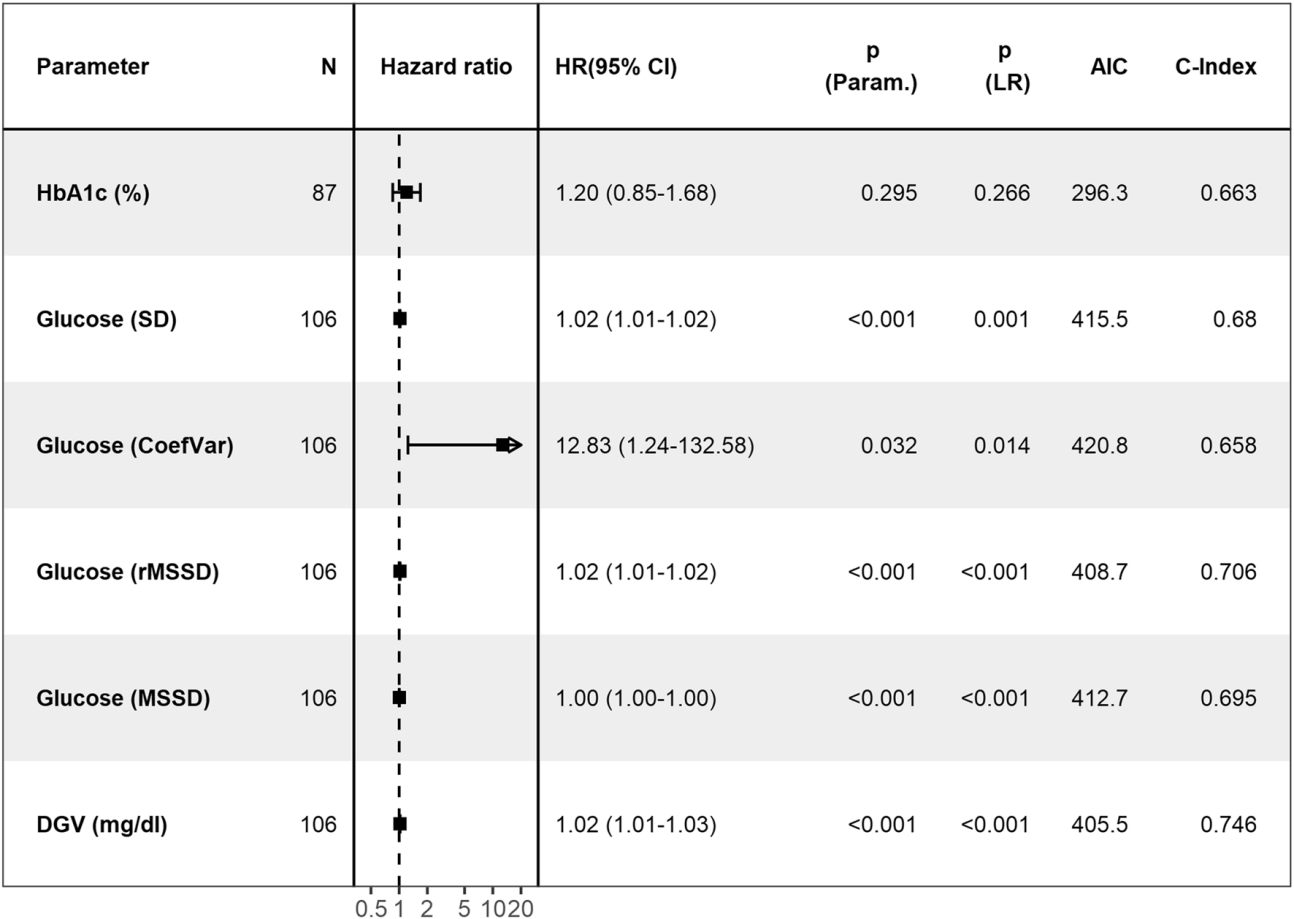
Comparison of HbA1c and variability metrics. Comparison of multivariable Cox-Proportional Hazard models, adjusted for age, sex and history of type 2 diabetes, for HbA1c and established variability metrics: standard deviation (SD), bias corrected Coefficient of Variation (CoefVar), Neuman’s (root) Mean Square of Successive Differences (rMSSD and MSSD) and median of the absolute successive difference between successive values (= DGV, daily glycaemic variability). Includes Hazard Ratio and 95% confidence intervals, *p*-value for the parameter and likelihood ratio test (LR), Akaike Information Criterion (AIC) and concordance index (C-Index).

Furthermore, we evaluated several established metrics of variability SD, CoefVar, MSSD, rMSSD, as well as DGV of FPG as continuous parameters of FPG variability in multivariable Cox-PH models. While these models include age, sex and history of T2D as a covariate for adjustment, neither were statistically significant in any of the models. Out of these variability metrics, we selected the statistically significant metric with the highest C-Index and lowest AIC, where median DGV outperformed all the other contenders (Fig. 2).

**Figure 3:**
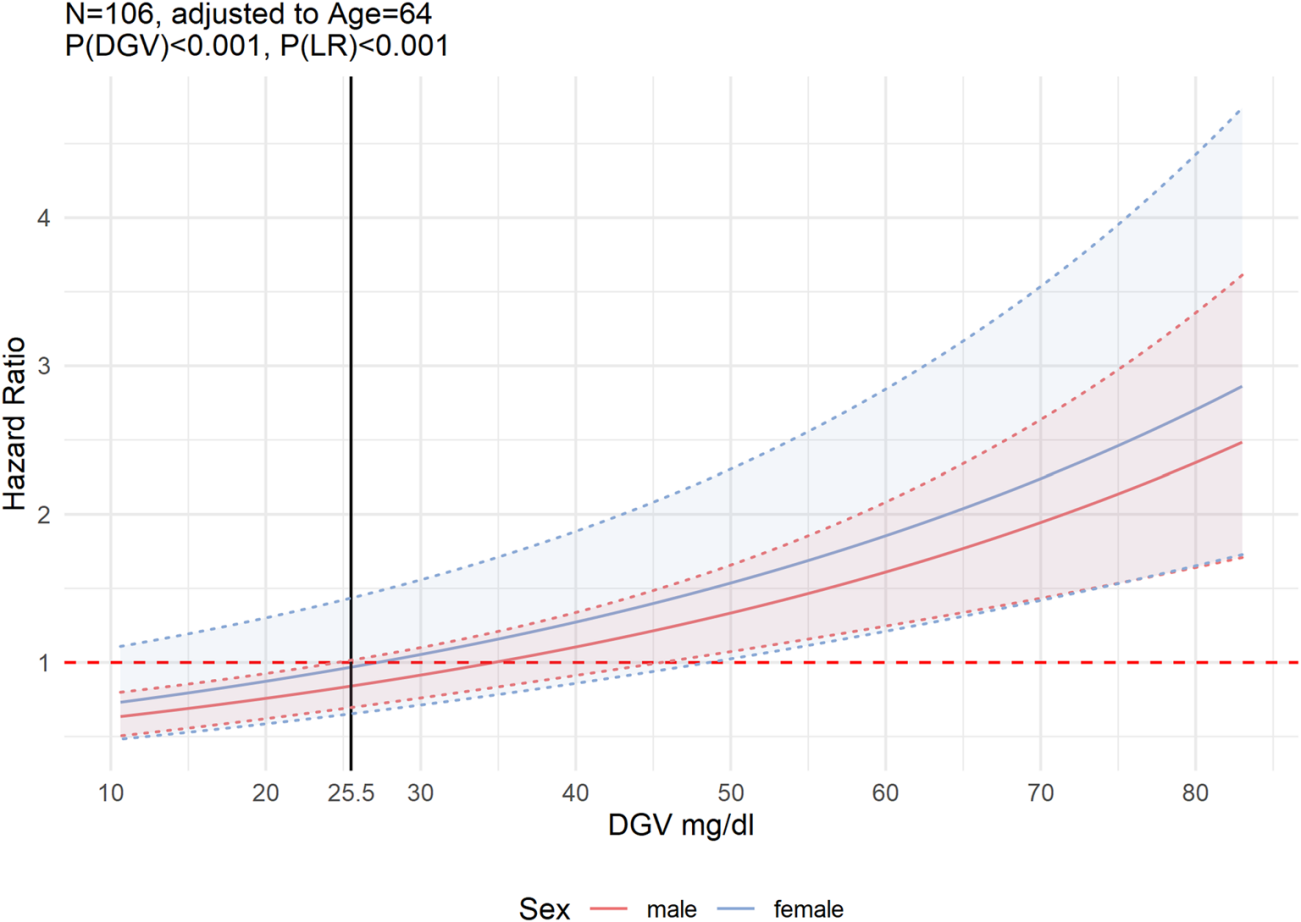
Relation between fatality risk and glucose variability. Continuous hazard ratios for fatality or non-survival were calculated by Cox proportional hazard models of daily glycaemic variability (DGV) adjusted for age and sex, including 95% CI for endpoint. The dashed red line notates a hazard ratio of 1.0, the solid black line the dichotomisation threshold, which we determined using a regression tree. The threshold of 25.5 mg/dl for DGV was used for our summary statistics (Tables 1 and 2), Kaplan–Meier curves (Fig. 4) and the adjusted DGV Cox proportional hazard model (Fig. 5).

Therefore, we created an age and sex adjusted Cox-PH model (LR(P) < 0.001) to demonstrate the change of HR by the range of DGV values. In this model, age and sex were not statistically significant in contrast to DGV (HR: 1.02 [95% CI 1.02–1.03], *P* < 0.001). The model showed a proportional increase in HR and at HR = 1 DGV was 34.63 mg/dl in males and 27.35 mg/dl in females (Fig. 3).

In the low DGV group, significantly more patients (n = 41 (69%)) survived to discharge compared to only 12 patients (25%) in the high DGV group. Furthermore, we analysed differences in clinical characteristics, comorbidities, laboratory parameters and medication on admission of patients in high and low DGV groups. There was no significant difference in age, gender, P/F ratio and likelihood of dialysis, ECMO and dexamethasone therapy among both groups (Table 2). Similarly, there were no statistically significant differences in symptoms reported at or prior to admission between both groups. However, patients in high DGV group were significantly more obese (Median [IQR]: 28.4 [26.3—31.1] vs. 31.2 [27.5—38.1], *P* = 0.01) and had a lower temperature on admission to the ICU (Median [IQR]: 38.1 [37.3—38.6] vs. 37.6 [36.9—38.1], *P* < 0.05). Comparing inflammatory markers on admission to the ICU, we found ferritin to be significantly lower in the high DGV group, whereas leukocytes, lymphocytes, CRP, PCT and IL-6 remained not significantly different between the two groups (Table 1).

**Table 2:** Outcome parameters of patients admitted with COVID-19 and ARDS to the Intensive Care Unit.

	N	All	DGV < 25.5 mg/dl	DGV ≥ 25.5 mg/dl	*P* value
		(N = 106)	(N = 59)	(N = 47)	
Endpoint at close-out: Survivor	106	53 (50·0%)	41 (69·5%)	12 (25·5%)	** < 0.01**
Hospitalisation (days)	106	32.5 (20.0–56.1)	38.0 (28.0–61.7)	23.0 (16.0–38.3)	** < 0.01**
ICU (days)	106	28.0 (16.0–44.3)	36.0 (21.5–58.7)	21.0 (10.3–31.8)	** < 0.01**
Ventilation (days)	106	25.0 (14.0–44.0)	34.0 (18.3–52.8)	20.0 (10.2–31.8)	** < 0.01**
ECMO therapy	106	30 (28·3%)	18 (30·5%)	12 (25·5%)	0.67
Dialysis	106	70 (66·0%)	34 (57·6%)	36 (76·6%)	0.06
Dexamethasone	106	43 (40·6%)	23 (39·0%)	20 (42·6%)	0.84
Glucose, median (mg/dl)	106	149.2 (135.0–168.1)	141.5 (129.0–151.8)	168.0 (148.9–186.8)	** < 0.01**
FPG, 3-day median (mg/dl)	106	155.0 (138.0–193.1)	146.0 (129.8–169.0)	185.5 (148.9–210.2)	** < 0.01**
DGV (mg/dl)	106	22.5 (15.9–33.0)	16.0 (13.0–20.0)	35.0 (29.5–52.3)	** < 0.01**
DGV, 3-day median (mg/dl)	106	31.0 (16.0–55.1)	22.0 (13.2–31.8)	42.0 (32.2–93.5)	** < 0.01**
Procalcitonin, median (ng/ml)	106	0.8 (0.3–2.6)	0.5 (0.2–1.5)	1.5 (0.6–4.5)	** < 0.01**

Numerical parameters are expressed as median (IQR) and categorical as N (%). The N column represents the number of non-missing values in each row to the right. *P*-values for differences between the two groups were tested using Fisher’s exact test for categorical variables and Kruskal–Wallis test for continuous variables. Bold indicates significant differences (*P* < 0.05). Parameters denoted with * are calculated over the first three days (“3-day”) or entire course of ICU admission. Daily glycaemic variability (DGV) is explained in the methods section.

**Figure 4:**
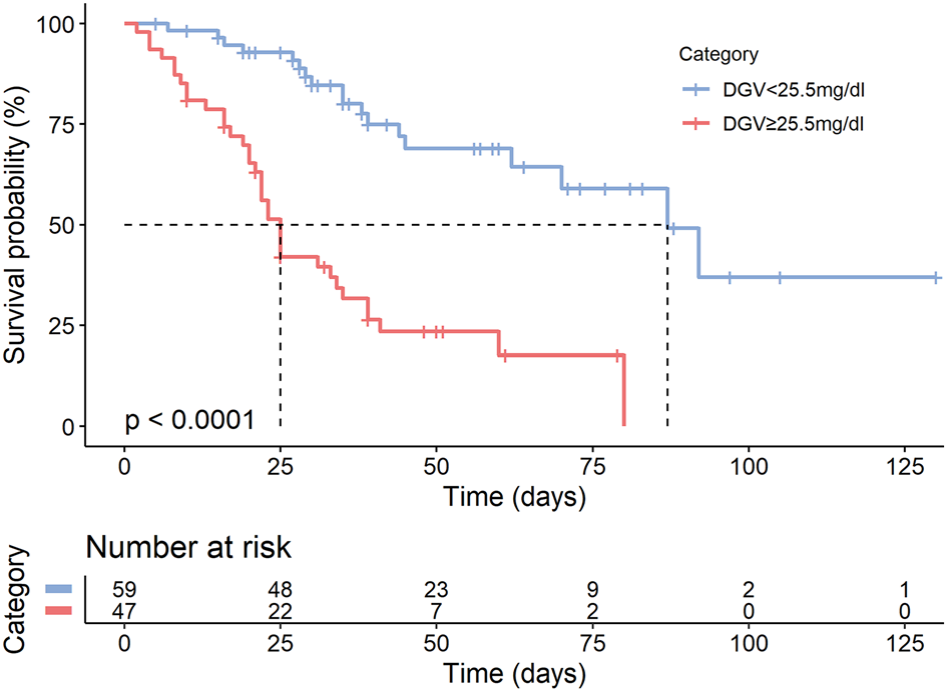
Kaplan–Meier Survival for ARDS Patients. Kaplan–Meier survival curves for patients with low (< 25.5 mg/dl) and high (≥ 25.5 mg/dl) daily glycaemic variability (DGV). Each cross on a curve represents a censored patient (survived to discharge), while each step in the curve represents a deceased patient. The x-axis represents the time in days to discharge. Survival curves were compared, and *p*-values calculated by log-rank test.

Additionally, Kaplan–Meier estimators showed a significantly (*P* < 0.0001) longer median survival of 87 days in the low DGV group in comparison to 25 days in the high DGV group (Fig. 4).

Based on these findings, we generated an unadjusted Cox-PH model for mortality in DGV (HR: 1.02, (P) < 0.001, LR(P) < 0.001). This model was still significant after adjusting for clinically predetermined confounders (HR: 1.016, (P) = 0.001, LR(P) < 0.001).

**Figure 5:**
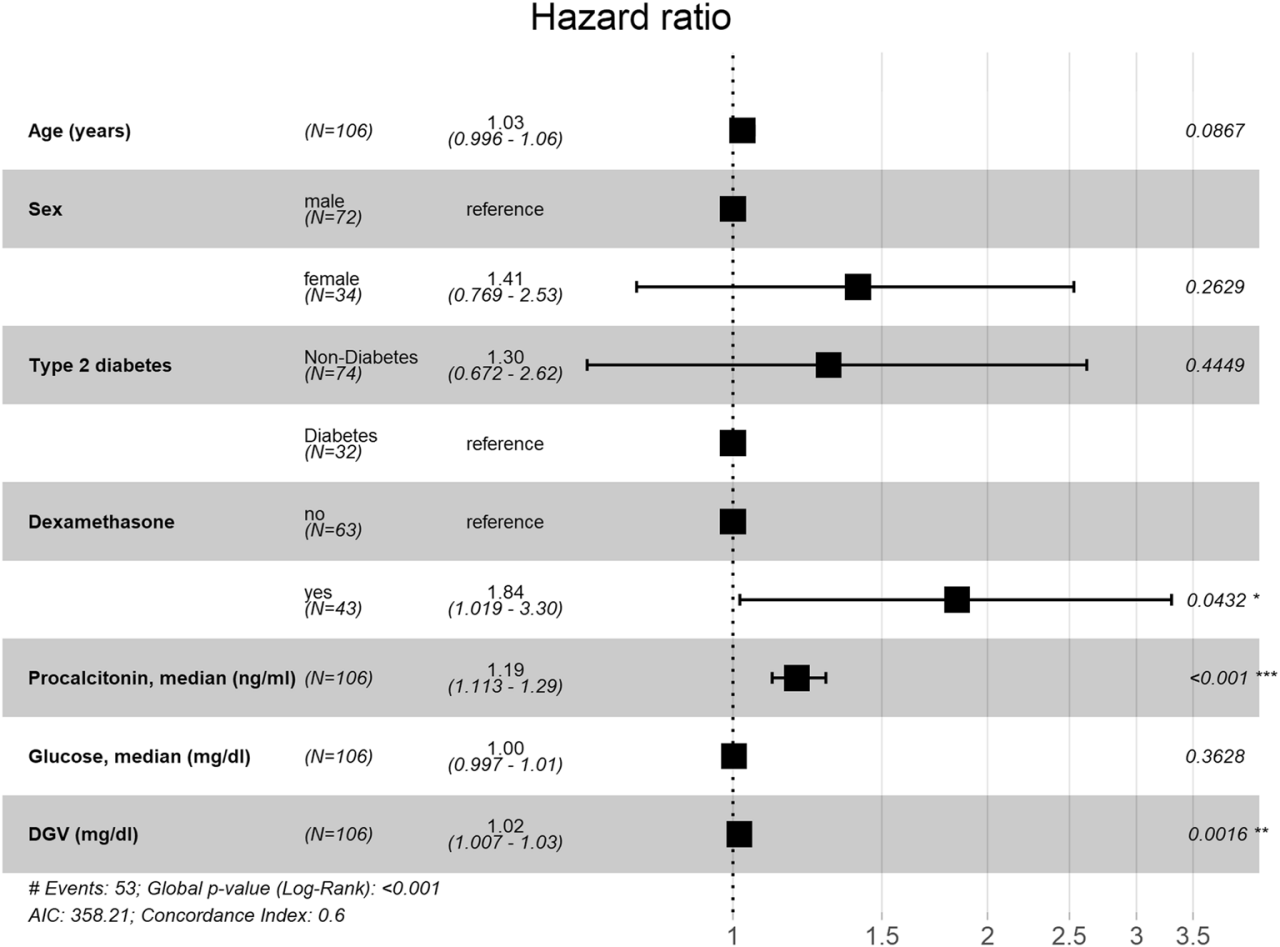
Adjusted hazard ratios for non-survival by DGV. Multivariable Cox proportional hazard model for median daily glycaemic variability (DGV) cut-off as a continuous covariate dichotomised at DGV ≥ 25.5 mg/dl, adjusted for clinically predetermined confounders: age, sex, type 2 diabetes status, dexamethasone treatment, median of procalcitonin, glucose and DGV during ICU admission.

In this model DGV, median PCT and treatment with dexamethasone were statistically significant (Fig. 5). However, median FPG levels did not show statistical significance, and collinearity between median FPG (VIF = 1.90) and DGV (VIF = 2.08) did not affect the model.

## Discussion

To our knowledge, this is the first study to compare different glycaemic variability metrics in patients with COVID-19 and ARDS and provide evidence that higher glycaemic variability is associated with higher mortality in these patients.

Previous cohorts in China, Germany, France, Italy, and the US have already established the high prevalence of T2D^5,23–26^ among COVID-19 patients admitted to the hospital. Even though a prior diagnosis of T2D certainly predisposes patients for hyperglycaemia, a recent review^27^ identified hyperglycaemia in up to 40% of ICU patients, where an estimated 80% of patients with hyperglycaemia had no prior diagnosis of T2D. We found a similar prevalence of hyperglycaemia in our study, albeit history of T2D was equally distributed in patients with hyperglycaemia.

Moreover, the CORONADO study^5^ previously reported that HbA1c levels on admission in patients hospitalised for COVID-19 were not associated with adverse outcome. Similarly, a meta-analysis^28^ did not find a statistically significant association between HbA1c and disease severity. Sánchez Díaz et al. further explored^29^ the effect of high HbA1c levels on mortality outcome in a study of 56 patients requiring invasive mechanical ventilation due to COVID-19 ARDS. They were able to show an increased mortality in univariable analysis when dichotomising patients with HbA1c ≥ 6.5%, which they could not replicate in multivariable adjusted models. Patients in this study also had fewer days of mechanical ventilation and days in the ICU and hospital than patients in our study. Moreover, Zhu et al.^30^ showed in a systematic review of nine clinical trials with 2577 subjects that patients dichotomised at HbA1c ≥ 7% were more likely to have a poor prognosis. However, this study included patients both with and without ARDS and could not show statistical significance when HbA1c was used as a continuous covariate in a multivariable model. Another systematic review^31^ explored HbA1c as both a continuous variable and a dichotomization factor in studies recruiting patients both with and without ARDS. Among four studies, they demonstrated a significant association of continuous HbA1c with a composite endpoint of COVID-19 related mortality or worsening of severity. However, when analysing mortality alone in data extracted from two of the four studies, they could no longer find a statistically significant association.

With respect to COVID-19, several early studies identified hyperglycaemia as a relevant factor for an adverse outcome. Both higher FPG levels at admission^32^ as well as at day 2–3 of admission^33^ and higher glycaemic range between FPG and two-hour postprandial glucose^34^ have been discussed.

At the time of inception of this analysis, there were no studies, which explored a possible association between inter-subject glycaemic variability and mortality outcome in COVID-19 patients with ARDS. Therefore, we evaluated several metrics of glycaemic variability and concluded that DGV, even after adjustment for confounders, is a significant metric in patients with adverse outcomes. Additionally, recently a few studies have been published that analysed individual metrics of glycaemic variability. For example, high inter-subject coefficient of variation and standard deviation have been associated with disease progression^35^ and in-ICU mortality^36^ in COVID-19.

The ESC Working Group for Atherosclerosis and Vascular Biology published a position paper^37^ underlining the significance of further research of molecular mechanisms of endothelial dysfunction and identified T2D as one of the risk factors.

A recent review^38^ of twelve studies about the association of elevated FPG and adverse outcome in hospitalised COVID-19 patients concluded that impaired glucose homeostasis appears to be a common and relevant factor of adverse outcome. They explored several possible underlying molecular mechanisms of disease progression mediated by elevated serum FPG. For example, they suggested that increased blood glucose levels increase the glucose concentrations in the alveolar surface liquid, which has an adverse effect on immune defence against pathogens. They concluded that this increases viral replication rate and reduces viral clearing, which combined with elevated glucose levels amplifies endothelial dysfunction.

Additionally, an in-vivo experimental study^39^ of twenty-two healthy and twenty-seven subjects with newly diagnosed T2D, reported that iatrogenic induction of rapid changes in FPG levels caused more endothelial dysfunction and oxidative stress than induction of constant hyperglycaemia.

This could explain why HbA1c levels did not correlate with adverse outcome in our adjusted models, since HbA1c levels primarily reflect the average blood glucose levels rather than high glycaemic variability. Furthermore, a high HbA1c level at admission represents the patient’s average blood glucose levels in the 8–12 weeks prior to admission, which might predispose patients to disease progression but does not reflect short term effects of high variability, which seem to modulate mortality outcome in the ICU.

## Limitations

This study was conceptualised as a retrospective subcohort study at a time where the SARS-CoV-2 pandemic had already reached parts of Germany and a disease cluster in Heinsberg, located 30 km north of our hospital in Aachen, had already developed. Therefore, some laboratory values had missing data points, which we outlined in the N column in all our tables. While some of the described parameters did not show significant differences between groups, we note the relatively small size of ICU patients with ARDS and COVID-19. Notably we found the majority of our sample to includes a high degree of severe COVID-19 cases, this could indicate a selection bias. We opted not to split our data in a training and validation dataset for model creation, due to the already small sample size, therefore a validation of our model in a different study population is warranted.

As a retrospective study, we did not perform a sample size calculation prior to the study or a post-hoc analysis^40^.

## Conclusion

The present study shows that high variability of FPG, not HbA1c, is significantly associated with an increased risk of all-cause mortality in severely ill COVID-19 patients with ARDS. This effect appears to be in line with similar research on glycaemic variability and possible mechanisms that contribute to increased mortality due to impaired glucose haemostasis. Our models suggest glycaemic variability is independent of clinically predetermined confounders and warrants further research in order to assess the predictive value. We suggest comparing these results in more generalised patient populations with ARDS both with and without COVID-19. However, these findings further emphasize the significance of monitoring FPG variability (DGV) in patients admitted to the ICU.

## Supplementary Information

Supplementary Information.

## Data Availability

The data underlying this article will be shared on reasonable request to the corresponding author.

## Abbreviations

AIC: Akaike Information Criteria
ARDS: Acute respiratory distress syndrome
BAL: Bronchoalveolar lavage
C-Index: Concordance Index
CoefVar: Coefficient of variation
COVID-19: Coronavirus disease
Cox-PH: Cox-proportional-hazard model
CRP: C-reactive protein
CT: Computed tomography
DGV: Daily glycaemic variability
ECMO: Extracorporeal membrane oxygenation
FPG: Fasting plasma glucose
HR: Hazard ratio
HbA1c: Glycated haemoglobin
ICU: Intensive care unit
IL-6: Interleukin 6
LR: Likelihood ratio
(r)MSSD: Neuman’s (root) mean square of successive differences
P/F ratio: Horowitz index
PCR: Polymerase-chain-reaction
PCT: Procalcitonin
SARS-CoV-2: Severe acute respiratory distress syndrome coronavirus 2
T2D: Type 2 Diabetes Mellitus
