# Supplementary material for: Glycaemic variability is associated with all-cause mortality in COVID-19 patients with ARDS, a retrospective subcohort study
Source: Sci Rep. 2022 Jun 14;12:9862. doi: 10.1038/s41598-022-13816-8 (PMC9194894; doi:10.1038/s41598-022-13816-8)
Supplement: Supplementary file 1 — Supplementary Information. [file 41598_2022_13816_MOESM1_ESM.docx]

# Supplementary Table 1: Diagnostic parameters and laboratory values on admission

|  | N | Overall | DGV<25.5mg/dl | DGV>=25.5mg/dl | P value |
| --- | --- | --- | --- | --- | --- |
|  |  | (N=106) | (N=59) | (N=47) |  |
| cycle threshold, S-gene | 40 | 24.0 (19.8—27.0) | 22.6 (18.3—25.5) | 25.6 (19.8—28.0) | 0.29 |
| Viral load | 67 |  |  |  | 0.59 |
| low |  | 9 (13·4%) | 4 (12·5%) | 5 (14·3%) |  |
| mild |  | 29 (43·3%) | 16 (50·0%) | 13 (37·1%) |  |
| high |  | 29 (43·3%) | 12 (37·5%) | 17 (48·6%) |  |
| Positive external SARS-CoV-2 PCR | 55 | 54 (98·2%) | 30 (100·0%) | 24 (96·0%) | 0.45 |
| Other viral pathogens, lavage | 68 | 31 (45·6%) | 18 (47·4%) | 13 (43·3%) | 0.81 |
| Fungal culture, lavage | 72 | 30 (41·7%) | 19 (46·3%) | 11 (35·5%) | 0.47 |
| Bacterial culture, lavage | 72 | 36 (50·0%) | 21 (51·2%) | 15 (48·4%) | 1.00 |
| Cellcount, lavage (10^4^/ml) | 68 | 14.8 (6.9—28.7) | 14.5 (8.5—27.8) | 15.6 (5.0—31.3) | 0.78 |
| Monocytes, lavage (%) | 68 | 55.5 (28.8—73.2) | 54.0 (27.7—71.3) | 61.0 (38.3—74.0) | 0.26 |
| Neutrophiles, lavage (%) | 68 | 38.0 (23.0—69.2) | 41.0 (25.7—71.0) | 34.0 (18.0—61.0) | 0.27 |
| Eosinophiles, lavage (%) | 68 | 0.0 (0.0—1.0) | 0.0 (0.0—0.0) | 0.0 (0.0—1.0) | 0.33 |
| Lymphocytes, lavage (%) | 66 | 2.0 (0.0—3.0) | 2.0 (0.0—3.0) | 2.0 (1.0—3.8) | 0.32 |
| Positive serum SARS-CoV-2 PCR | 48 | 25 (52·1%) | 15 (53·6%) | 10 (50·0%) | 1.00 |
| Positive stool SARS-CoV-2 PCR | 26 | 13 (50·0%) | 6 (40·0%) | 7 (63·6%) | 0.43 |
| Positive urine SARS-CoV-2 PCR | 44 | 5 (11·4%) | 3 (12·5%) | 2 (10·0%) | 1.00 |
| Positive respiratory PCR, Day 1 | 92 | 82 (89·1%) | 43 (86·0%) | 39 (92·9%) | 0.34 |
| Positive respiratory PCR, Day 7 | 73 | 50 (68·5%) | 27 (65·9%) | 23 (71·9%) | 0.62 |
| Positive respiratory PCR, Day 14 | 60 | 26 (43·3%) | 19 (47·5%) | 7 (35·0%) | 0.42 |
| Leukocytes (/µl) | 106 | 10.0 (7.4—13.6) | 10.0 (7.7—13.5) | 9.7 (7.1—14.5) | 0.96 |
| Hemoglobin (g/dl) | 106 | 11.4 (9.7—12.5) | 11.4 (9.9—12.7) | 11.4 (9.4—12.2) | 0.25 |
| Thrombocytes (/µl) | 106 | 245.0 (176.8—317.9) | 237.0 (191.0—340.3) | 255.0 (153.2—313.5) | 0.73 |
| Neutrophile_ICU | 19 | 87.0 (84.3—91.6) | 87.0 (83.8—91.8) | 87.0 (83.1—91.8) | 1.00 |
| Lymphocytes (%) | 90 | 6.0 (3.7—10.6) | 6.0 (3.4—10.9) | 6.3 (3.8—10.6) | 0.70 |
| Lymphocytes (/µl) | 90 | 0.7 (0.3—1.1) | 0.7 (0.3—1.1) | 0.7 (0.5—1.0) | 0.92 |
| Monozyten_ICU | 28 | 3.6 (2.3—6.0) | 4.1 (3.0—6.0) | 3.0 (2.0—8.0) | 0.90 |
| Eosinophiles (%) | 88 | 0.0 (0.0—0.6) | 0.0 (0.0—0.8) | 0.0 (0.0—0.5) | 0.36 |
| Basophile_ICU | 19 | 0.2 (0.0—0.3) | 0.2 (0.1—0.3) | 0.2 (0.0—0.3) | 0.45 |
| INR (ratio) | 106 | 1.2 (1.1—1.3) | 1.2 (1.1—1.3) | 1.2 (1.1—1.3) | 0.05 |
| aPTT (sec) | 106 | 29.9 (27.6—34.4) | 29.9 (27.5—33.7) | 29.9 (28.2—38.2) | 0.58 |
| Antithrombin III (%) | 25 | 80.0 (74.0—99.0) | 80.5 (74.0—103.3) | 79.0 (59.0—90.0) | 0.59 |
| Fibrinogen (mg/dl) | 31 | 571.0 (404.2—684.0) | 575.0 (405.9—688.7) | 439.0 (384.5—651.3) | 0.36 |
| D-dimer (µg/l) | 79 | 2252.0 (1237.8—7168.5) | 3525.0 (1389.5—7244.5) | 1614.5 (755.6—5688.1) | 0.09 |
| TZ (sec) | 54 | 15.6 (14.4—17.9) | 15.3 (14.0—17.3) | 16.2 (14.4—18.0) | 0.62 |
| Sodium (mmol/l) | 104 | 140.0 (137.0—144.0) | 140.0 (137.0—144.8) | 140.0 (136.7—144.0) | 0.55 |
| Potassium (mmol/l) | 104 | 4.5 (4.0—5.2) | 4.5 (4.0—5.0) | 4.4 (4.0—5.4) | 0.85 |
| Phosphate (mmol/l) | 47 | 1.2 (0.9—1.5) | 1.2 (0.8—1.4) | 1.3 (1.1—1.6) | 0.15 |
| Albumine (mg/dl) | 101 | 2.7 (2.2—3.1) | 2.7 (2.3—3.2) | 2.7 (2.2—3.0) | 0.28 |
| Bilirubin, total (mg/dl) | 106 | 0.6 (0.4—0.9) | 0.6 (0.4—0.9) | 0.6 (0.3—0.9) | 0.70 |
| Bilirubin, direct (mg/dl) | 56 | 0.4 (0.3—1.5) | 0.4 (0.3—0.6) | 1.5 (0.3—2.0) | **<0.05** |
| ASAT (U/l) | 106 | 56.5 (35.0—89.0) | 58.0 (35.2—88.5) | 51.0 (35.3—90.2) | 0.87 |
| ALAT (U/l) | 105 | 37.0 (25.0—58.3) | 39.0 (26.2—61.5) | 36.0 (24.8—51.3) | 0.33 |
| gamma-GT (U/l) | 103 | 77.0 (40.2—146.8) | 66.0 (39.7—131.7) | 88.5 (40.9—152.4) | 0.38 |
| AP (U/l) | 103 | 75.0 (53.0—101.3) | 74.0 (52.3—92.0) | 77.0 (54.8—116.4) | 0.26 |
| LDH (U/l) | 105 | 451.0 (369.7—627.3) | 437.0 (369.2—636.3) | 463.0 (374.6—604.8) | 0.71 |
| CK (U/l) | 106 | 171.5 (60.0—412.0) | 167.0 (59.2—390.8) | 176.0 (84.2—430.0) | 0.88 |
| CK-MB activity (U/l) | 53 | 23.0 (17.3—31.3) | 26.0 (20.0—31.3) | 19.5 (13.0—39.9) | 0.28 |
| hsTroponin-T (pg/ml) | 71 | 28.0 (14.0—49.8) | 25.0 (13.9—50.0) | 28.0 (14.7—52.0) | 0.74 |
| NTproBNP (pg/ml) | 62 | 656.1 (240.8—2829.8) | 691.3 (284.7—3098.8) | 557.4 (182.3—2918.7) | 0.65 |
| Urea (mg/dl) | 106 | 58.5 (34.8—90.2) | 52.0 (32.2—70.7) | 68.0 (38.0—110.8) | 0.07 |
| Creatinine (mg/dl) | 106 | 1.1 (0.8—1.9) | 1.0 (0.8—1.5) | 1.2 (0.9—2.4) | 0.15 |
| CRP (high-sense) (mg/l) | 97 | 169.0 (113.6—287.3) | 172.7 (131.4—275.4) | 160.0 (86.8—300.7) | 0.50 |
| Procalcitonin (ng/ml) | 106 | 0.5 (0.2—1.7) | 0.4 (0.2—1.2) | 0.6 (0.2—1.9) | 0.31 |
| Ferritin (ng/ml) | 56 | 1389.5 (823.9—2641.0) | 1690.0 (1054.0—2792.7) | 1020.5 (662.2—2057.8) | **<0.05** |
| pH | 95 | 7.4 (7.3—7.4) | 7.4 (7.3—7.5) | 7.3 (7.3—7.4) | 0.16 |
| arterial CO2 partial-pressure (mmHg) | 95 | 46.5 (35.5—60.4) | 46.3 (36.7—59.1) | 46.5 (34.0—63.2) | 0.90 |
| arterial bicarbonate (mmol/l) | 95 | 25.5 (23.1—29.9) | 26.4 (23.3—30.0) | 25.5 (22.9—28.8) | 0.63 |
| arterial Base-Excess | 95 | 0.4 (-2.2—3.9) | 0.5 (-1.9—4.1) | 0.4 (-2.2—2.9) | 0.41 |
| arterial oxygen partial-pressure (mmHg) | 95 | 75.8 (60.9—92.9) | 75.2 (60.9—90.5) | 80.8 (61.3—101.8) | 0.38 |
| arterial oxygen saturation (%) | 95 | 94.6 (90.9—96.9) | 94.5 (91.3—96.7) | 94.9 (90.2—97.5) | 0.57 |
| Methemoglobin (%) | 75 | 1.0 (0.8—1.2) | 0.9 (0.7—1.1) | 1.0 (0.8—1.3) | 0.27 |
| Carboxyhemoglobin (%) | 84 | 0.9 (0.7—1.2) | 0.9 (0.7—1.2) | 0.9 (0.6—1.2) | 0.78 |
| Lactate (mmol/l) | 106 | 1.3 (0.9—1.9) | 1.2 (0.8—1.9) | 1.3 (0.9—1.9) | 0.62 |
| Cystatin-C (mg/l) | 16 | 1.3 (1.1—2.2) | 1.2 (1.1—1.7) | 4.1 (1.6—4.2) | 0.11 |
| Myoglobin (µg/l) | 21 | 110.5 (58.5—360.2) | 109.6 (49.1—253.6) | 283.7 (75.6—810.1) | 0.28 |
| Copeptin (pmol/l) | 16 | 17.7 (8.5—48.1) | 14.3 (7.4—33.1) | 53.7 (23.8—77.2) | 0.12 |
| Aldosterone (pg/ml) | 16 | 50.1 (37.3—206.5) | 43.4 (34.7—96.1) | 229.0 (127.3—246.5) | 0.09 |
| sIL-2R (U/ml) | 24 | 1839.5 (896.2—2170.2) | 1534.5 (889.5—2001.2) | 2723.0 (1751.3—5679.6) | 0.10 |
| IL-6 (pg/ml) | 84 | 142.9 (77.9—325.5) | 133.1 (67.0—338.0) | 160.9 (95.1—294.3) | 0.54 |
| TNFalpha (pg/ml) | 25 | 14.1 (10.4—20.2) | 12.1 (11.0—18.2) | 29.4 (6.8—55.5) | 0.43 |
| B-Lymphocyte count (/µl) | 12 | 144.0 (63.8—233.7) | 114.0 (51.0—228.7) | 210.0 (95.0—254.2) | 0.52 |
| B-Lymphocyte fraction (%) | 12 | 24.0 (13.7—33.6) | 25.0 (19.7—40.0) | 16.0 (12.7—19.3) | 0.16 |
| T-Lymphocyte count (/µl) | 12 | 291.5 (218.8—790.4) | 275.0 (191.0—731.3) | 777.0 (343.7—1608.7) | 0.23 |
| T-Lymphocyte fraction (%) | 12 | 62.5 (54.0—72.8) | 57.0 (49.7—69.7) | 74.0 (59.8—79.8) | 0.19 |
| T-Helper count (/µl) | 12 | 213.0 (146.2—513.5) | 181.0 (137.0—404.0) | 578.0 (213.0—918.0) | 0.23 |
| T-Helper fraction (%) | 12 | 42.5 (29.2—50.0) | 42.0 (27.7—50.0) | 45.0 (33.3—53.3) | 0.52 |
| T-Killer count (/µl) | 12 | 91.0 (39.8—284.0) | 59.0 (30.0—193.7) | 179.0 (119.8—650.7) | 0.12 |
| T-Killer fraction (%) | 12 | 16.0 (9.8—25.2) | 11.0 (8.3—21.3) | 24.0 (18.2—32.3) | 0.16 |
| T-Helper to T-Killer ratio (%) | 12 | 2.5 (1.3—4.1) | 2.6 (1.9—4.8) | 1.3 (1.3—2.9) | 0.40 |
| NK-cell count (/µl) | 12 | 63.0 (46.5—105.8) | 56.0 (42.7—75.0) | 122.0 (72.8—147.8) | 0.14 |
| NK-cell fraction (%) | 12 | 8.5 (7.0—20.6) | 9.0 (7.0—20.3) | 7.0 (6.2—23.7) | 0.64 |
| Urine-albumine (mg/l) | 15 | 51.0 (18.2—176.3) | 37.0 (18.9—266.8) | 103.0 (3.7—339.3) | 0.90 |
| Urine-creatinine (mg/dl) | 15 | 63.9 (15.5—116.5) | 107.5 (63.6—154.8) | 14.6 (9.8—26.2) | **0.01** |
| ACE (U/l) | 26 | 21.5 (12.2—30.8) | 20.2 (10.6—31.7) | 24.2 (12.8—29.6) | 0.98 |

Numerical parameters are expressed as median(IQR) and categorical as N(%). The N column represents the number of non-missing values in each row to the right. P-values for differences between the two groups were tested using Fisher’s exact test for categorical variables and Kruskal-Wallis test for continuous variables. Bold indicates significant differences (P<0.05).
